# Magnetic resonance and bioluminescence imaging of macrophage homing to experimental abdominal aortic aneurysms

**DOI:** 10.1186/1532-429X-13-S1-P369

**Published:** 2011-02-02

**Authors:** Monica M Dua, Noriyuki Miyama, Geoffrey M Schultz, Hisanori Kosuge, Masahiro Terashima, Laura J Pisani, Ronald L Dalman, Michael V McConnell

**Affiliations:** 1 Stanford University, Stanford, CA, USA

## Introduction

Macrophage infiltration is a prominent feature of abdominal aortic aneurysm (AAA) progression. We used a multimodality imaging approach - magnetic resonance (MRI) and bioluminescence (BLI) - to study macrophage homing and accumulation in experimental AAA disease.

## Purpose

Employ cellular imaging methods to track and image macrophages in AAA disease.

## Methods

Murine AAAs were created via intra-aortic infusion of porcine pancreatic elastase and aortic size was monitored by ultrasound on post-op day (POD) 3, 7, and 14. Mice were imaged after injection of prepared peritoneal macrophages. For BLI, macrophages were from transgenic mice expressing luciferase, with injection on POD 1 and imaging at POD 3, 7, and 14 by in situ BLI (AAA N=17, sham control N=15). For MRI, macrophages were labeled with iron oxide particles (Feridex), with injection on POD 13 and imaging at POD 14 by in vivo 7 T MRI (AAA N=8, sham N=5). Mice were sacrificed after imaging for histological analysis.

## Results

**Figure 1:**
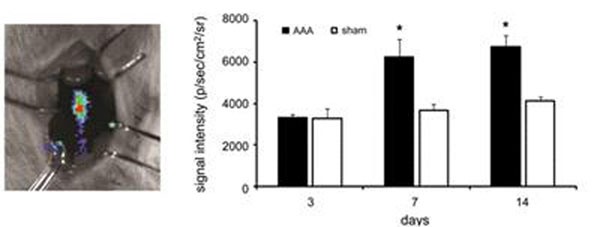
Shows in situ BLI of AAA (left), with significant macrophage BLI signal detected at POD 7 and 14.

**Figure 2:**
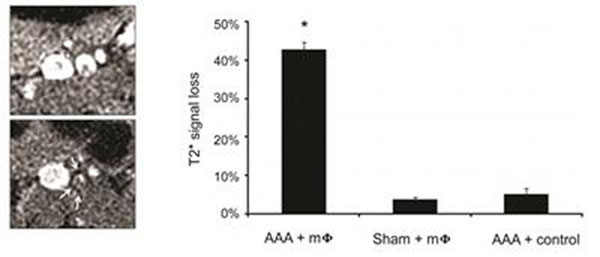
Shows MRI of AAA pre/post injection, with concentric T2* signal loss post-injection (left, arrows), which was not seen with sham operation or cell-free controls (right).

In situ BLI (Fig 1) demonstrated a significant increase in macrophage signal in the AAA by POD 7 (AAA: 6251 ± 852 vs. sham: 3669 ± 281 photon/sec/cm2/sr, P < 0.05), persisting at POD 14 (6754 ± 532 vs. 4135 ± 184 photon/sec/cm2/sr, P < 0.01), which correlated with macrophage number and aortic diameter (r=0.62-0.68, P < 0.05 for both). In vivo 7T MRI (Fig 2) demonstrated T2* signal loss at POD 14 in the AAA and not in sham (AAA: 43 ± 2% vs. sham: 4 ± 0.7%, P < 0.01). This finding was specific for Feridex-labeled macrophages, as injection of cell-free Feridex solution (N=3) did not induce T2* signal loss in AAAs. Immunohistochemistry and Prussian blue staining confirmed the presence of injected macrophages in the AAA.

## Conclusions

MRI detects homing of macrophages to AAA in vivo, while in situ BLI can follow macrophage accumulation over time. Thus, MRI and BLI provide complementary approaches to track macrophage biology in experimental AAAs.

